# Societal Perspectives and Real-World Cost-Effectiveness: Expanding the Scope of Health Economics Inquiry

**DOI:** 10.3390/curroncol30010018

**Published:** 2022-12-23

**Authors:** Christopher J. Longo

**Affiliations:** 1 Health Policy and Management, DeGroote School of Business, McMaster University, Burlington, ON L7L 5R8, Canada; clongo@mcmaster.ca; 2 Dalla Lana School of Public Health, University of Toronto, Toronto, ON L8S 4L8, Canada

Economic evaluations of health technologies for cancer are frequently seen in the literature, but not all economic perspectives have the same frequency. Although a health system perspective is commonly used to address the needs of the payer, these analyses tend to ignore the broader economic impact on patients and their families. In order to understand these broader perspectives, economic evaluations need to include other effects, such as patient out-of-pocket costs, travel costs, lost income for patients and caregivers, and potentially their combined impacts on quality of life.

Similarly, the majority of cost-effectiveness analyses in cancer are based on clinical trials, which typically lack generalizability. Additional analyses that examine cost-effectiveness using expanded methods and/or in real-world settings by using government and private payer databases or modeling techniques allow readers to understand whether trial-based cost-effectiveness aligns with the results seen after technology approval. These broader perspectives thereby provide a more generalizable outcome that are particularly useful for government and private payor decision makers. I will now provide an overview of the Special Issue papers that discuss these foci in greater detail.

The first collection of articles touches on the societal perspective from a variety of angles including an examination of human capital versus friction methods to determine the valuation of premature mortality in Europe for 2020 [1]; the economic burden of cancer in Canada from a societal perspective in 2021 [2]; the health-related out-of-pocket costs of cancer in Canada using linked-datasets with observations [3]; and finally a discussion of the patient financial toxicity and the possible link to outcomes [4].

Hanly et al. [1] posited that the friction cost approach (FCA) provides a more conservative estimate on the overall economics of lost productivity when the unemployed can replace those lost from the workplace after a period of training (the friction period). These estimates in cancer are 11.1 to 64.5 times lower than the human capital approach, depending on the European country examined. Hanly et al. decided to examine European countries across a variety of cancer sites using consistent methodology for premature mortality to better understand the variance using the GLOBOCAN 2020 database. These analyses highlight that methods of measuring lost productivity can provide very different conclusions about the economic impact, and encourage us to more closely examine which approaches are most appropriate.

Garaszczuk et al. [2] examined the economic burden of cancer from a societal perspective in Canada using the Canadian OncoSim microsimulation model across 31 cancers. It uses Canadian Cancer Registry data and Statistics Canada demography data. This examination revealed that 30% of CAD 26.2 billion was borne by patients and their families (CAD 7.8 billion), with the largest portion of this burden occurring in the first year after diagnosis (CAD 4.8 billion), and included out-of-pocket costs, indirect costs, and time costs. These analyses help to provide a more comprehensive estimate of the total economic burden of cancer in Canada.

Essue et al. [3] examined household out-of-pocket costs compared to a matched control group. They used national databases and found that, for the most part, there was no statistically significant difference between groups, although values were generally higher for cancer patients and approached statistical significance in colorectal cancer. They noted that many of the households exceeded a year after treatment, and acknowledged that other direct survey data suggest that costs are higher during the active treatment portion of their care. This study highlights the value in having a control group, thus highlighting that costs measured using other methods can ignore the underlying base rate of out-of-pocket costs related to “usual care”. Hence, some of these survey-based examinations may measure both cancer-related and non-cancer-related costs in aggregate.

Longo [4] examined the impact of financial toxicity on both patient’s quality of life (QoL) and their decisions to forego or delay care. He highlights published work suggesting that as costs rise for care (including the cost of newer drugs), evidence suggests a greater risk for negative impacts on QoL and decisions to forego or delay care. Six studies showed a decreased QoL for patients with financial challenges. An additional three studies suggested that financial toxicity was linked to poorer overall survival. His paper called for further research to more accurately and consistently quantify these effects so that they can be included in economic analyses.

The second collection of articles on real-world cost-effectiveness that progresses from a typical clinical trial with some minor adjustments to improve generalizability [5] includes a systematic review that included a subgroup of real world cost-effectiveness data [6] and a rapid review of real-world cost-effectiveness studies in Canada [7]. 

Bao et al. [5] reported results from two clinical trials, representing a typical approach in relation to economic evaluation in cancer research. A comparison of anti-HER2 second-line agents pyrotinib and lapatinib used a partitioned survival model with three health states (PFS, PD, and death). They included one-way sensitivity analyses presented as tornado diagrams, as well as a probabilistic analysis using 1000 Monte Carlo simulations. One portion of this analysis in relation to real-world cost-effectiveness involves the modeling of adjustments in product price to improve cost-effectiveness for pyrotinib (Study A). The authors acknowledge some of the limitations including the use of PFS and OS data from a phase II trial, which often differs from later-stage studies.

Yanev et al. [6] undertook a systematic review of metastatic hormone-sensitive (mHSPC) and non-metastatic castration-resistant (nmCRPC) prostate cancer. They examined 44 studies, of which 7 were real-world cost-effectiveness studies. This paper identified the most effective strategies in these populations based on clinical trial data, but added a brief analysis of the seven real-world data studies. Unfortunately, these seven real-world studies were all cost-effectiveness studies and none were comparative in nature, with six of the seven completed in the US and one in Sweden.

Guggenbickler et al. [7] undertook a rapid review of real-world cost-effectiveness analyses of cancer interventions in Canada. This paper highlights these studies, as well as the “real-world evidence working group” currently in existence in Canada. These studies rely on provincial cancer agencies and Ministries of Health across a number of provinces. After the title, abstract, and full-text review, 22 of the original 206 identified studies met inclusion criteria. Most of these studies used sample sizes between 1000 and 2000 patients, but some exceeded 10,000. These studies included drug comparisons (59%), with the balance on screening (32%), preventing recurrence (4.5%) and assistive surgery (4.5%). Median ICERs were higher for drug studies, those without models (person level data), those without QALYs, and those after 2017, although not statistically significant, excepting those without models.

This Special Issue summarizes both societal perspectives in economic evaluation and real-world cost-effectiveness. These are still areas where a paucity of data exists, but there are some encouraging signs. A Canadian study on real-world cost-effectiveness suggests that these types of analyses have become more common over the past 10 years, and are needed to ensure that investments in new technology are delivering the value promised by RCT data. The integration of more societal perspectives seems to be lagging behind a bit, perhaps influenced by the need to inform decision makers mostly focused on government budgets. However, some good work on societal cost burdens including out-of-pocket costs is encouraging. I am hopeful that this type of forum will make more decision makers aware of the societal perspective and the value it brings to the table by highlighting the impacts on lost work and out-of-pocket costs which are real problems for patients and their families, most notably during their treatment but still evident years after treatment is complete. This approach recognizes that value goes beyond clinical effectiveness and even traditional cost-effectiveness to embrace a more multi-dimensional view of value, as described by Lakdawalla et al. [8].

## References

[1] Hanly P., Ortega-Ortega M., Soerjomataram I. (2022). Cancer premature mortality costs in Europe in 2020: A comparison of the human capital approach and the friction cost approach. Curr. Oncol..

[2] Garaszczuk R., Yong J.H.E., Sun Z., de Oliveira C. (2022). The economic burden of cancer in Canada from a societal perspective. Curr. Oncol..

[3] Essue B.M., Oliveira C.d., Bushnik T., Fung S., Hwee J., Sun Z., Navas E.G., Yong J.H.E., Garner R. (2022). The burden of health-related out-of-pocket cancer costs in Canada: A case-control study using linked data. Curr. Oncol..

[4] Longo C.J. (2022). Linking intermediate to final “real-world” outcomes: Is financial toxicity a reliable predictor of poorer outcomes in cancer. Curr. Oncol..

[5] Bao Y., Zhang Z., He X., Cai L., Wang X., Li X. (2022). Cost-effectiveness of pyrotinib plus capecitabine versus lapa-tinib plus capecitabine for the treatment of HER-2 positive metastatic breast cancer in China: A scenario analysis for health insurance coverage. Curr. Oncol..

[6] Yanev I., Gatete J., Aprikian A.g., Guertin J.R., Dragomir A. (2022). The health economics of metastatic hormone-sensitive and non-metastatic castration-resistant prostate cancer—A systematic literature review with application to the Canadian context. Curr. Oncol..

[7] Gluggenbickler A.M., Barr H.K., Hoch J.S., Dewa C.S. (2022). Rapid review of real-world cost-effectiveness analyses of cancer interventions in Canada. Curr. Oncol..

[8] Lakdawalla D.N., Doshi J.A., Garrison L.P., Phelps C.E., Basu A., Danzon P.M. (2018). Defining elements of value in health care—A health economics approach: An ISPOR special task force report. Value Health.

